# Surgical castration in dogs: does the incision approach influence postoperative recovery?

**DOI:** 10.18849/ve.v7i4.587

**Published:** 2022-12-29

**Authors:** Ariel Brunn+

**Keywords:** CANINE, CASTRATION, COMPLICATIONS, ORCHIDECTOMY, PAIN, SCROTUM, STERILISATION, SURGERY

## Abstract

**PICO question:**

In male dogs undergoing surgical castration, does a pre-scrotal approach in comparison to a scrotal approach lead to a superior recovery, in terms of duration of postoperative pain and/or reduced post-operative complications?

**Clinical bottom line:**

**Category of research:**

Treatment.

**Number and type of study designs reviewed:**

Two prospective clinical trials were critically appraised.

**Strength of evidence:**

Weak.

**Outcomes reported:**

Woodruff et al. (2015) evaluated postoperative recovery in 206 dogs following surgical castration using a scrotal incision in comparison to 231 dogs using a pre-scrotal approach. Complications observed in order of frequency, included: incisional swelling; haemorrhage; pain; and self-trauma, however, apart from self-trauma, complications were not influenced by incision location. Dogs castrated using a scrotal approach had reduced odds of self-trauma (OR: 0.51, P = 0.04, 95% CI 0.27–0.97). Moreover, mean duration of surgery was faster for the scrotal versus the pre-scrotal approach (3.6 minutes, P<0.01, 95% CI 3.38–3.82 versus 5.1 minutes, 95% CI 4.86–5.41).

Miller et al. (2018) evaluated complication rates following open or closed castration using a scrotal approach in 400 shelter dogs under the age of 6 months. Complications involving intra-operative bleeding were not observed, while marginal rates of post-operative events were reported, including peri-incisional dermatitis (2.3%), skin bruising (1.0%), and swelling (0.3%). No self-trauma or rescue analgesia was recorded. In comparing surgical time, the mean duration was 1 minute ± 0.2 minutes in dogs undergoing scrotal surgery, in comparison to canine patients undergoing the same procedure using a pre-scrotal approach, where the mean duration was 3.5 minutes ± 0.4 minutes.

**Conclusion:**

The outcomes of these two studies imply that a scrotal incisional approach in canine castration is at least no worse in the first 24 hours than a traditional pre-scrotal approach and may also reduce mean duration of surgery. However, limitations to the evidence do not permit a firm conclusion and it also remains unclear whether these advantages persist in the longer postoperative period. Further research is needed to confirm initial findings suggested here.

How to apply this evidence in practice

The application of evidence into practice should take into account multiple factors, not limited to: individual clinical expertise, patient’s circumstances and owners’ values, country, location or clinic where you work, the individual case in front of you, the availability of therapies and resources.

Knowledge Summaries are a resource to help reinforce or inform decision making. They do not override the responsibility or judgement of the practitioner to do what is best for the animal in their care.

## Clinical scenario

You have agreed to provide elective neutering for a local animal shelter. There are a number of male dogs that need to be castrated prior to their imminent adoption and a colleague suggests you consider using the high-quality high-volume method of scrotal castration. Since you want to be certain that the technique you use is based on the best available evidence, you decide to review if postoperative pain and complication rates using a scrotal incisional approach are equivalent to the more familiar method of a pre-scrotal incision that you use on your client-owned patients.

## The evidence

Following a literature search in two databases, PubMed and CAB Abstracts, two papers met inclusion criteria for this summary. Both studies evaluated postoperative complications following orchidectomy via direct scrotal access, as well as measuring surgical duration in comparison to traditional castration methods.

## Summary of the evidence

**Woodruff et al. (2015):** 

Population:	Healthy male dogs older than 6 months with a body weight ranging from 3–60 kg (average 17 kg) were selected from five shelters associated with Mississippi State University (MSU) and the Humane Alliance (HA) in North Carolina. Cryptorchid dogs were excluded.
Sample size:	437 dogs in total.
Intervention details:	Random allocation of dogs into two treatment groups: 206 underwent castration with a pre-scrotal incision and 231 underwent castration with a scrotal incision. Dogs in each group were anaesthetised with intravenous (IV) butorphanol (0.35 mg/kg); ketamine (3.5 mg/kg); and dexmedetomidine (17.5 ug/kg) combination. Preoperative analgesia was given as subcutaneous carprofen (4.4. mg/kg). Dogs from both groups underwent identical surgical preparation of the scrotum and pre-scrotal area with a chlorhexidine scrub prior to being moved into theatre. Dogs were placed in dorsal recumbency and aseptically draped. Dogs in the group with pre-scrotal access had a skin incision made cranial to the scrotum and a closed method used for castration. The incision was closed using an interrupted intradermal pattern (2-0 polyglactin 910 suture). Dogs in the group with scrotal access had a midline incision made ventrally in the scrotum and a closed method of castration used, as per the pre-scrotal group. The incision was left open with placement of a single subcutaneous suture (2-0 polyglactin 910 suture). Post-operative complications were monitored by shelter employees or private owners.
Study design:	Randomised positive control multi-site clinical trial.
Outcome Studied:	Haemorrhage; pain; self-trauma for 24 hours; swelling at incision site after 2, 4, 6, 24, 48, 72 hours. Duration of surgery.
Main Findings(relevant to PICO question):	Self-trauma was observed in 54 dogs in total (pre-scrotal incision group: 34 dogs; scrotal incision group: 20 dogs). The odds of self-trauma were reduced in dogs with a scrotal incision (OR: 0.51, P = 0.04, 95% CI 0.27­–0.97). Haemorrhage, pain and swelling at any time after surgery were not significantly different between the two interventions. Length of surgery was shorter on average for the scrotal approach group (3.6 minutes, P<0.01, 95% CI 3.38–3.82 versus 5.1 minutes for the pre-scrotal group, 95% CI 4.86–5.41) (measured at one site only – MSU).
Limitations:	Postoperative monitoring was done by shelter staff in Mississippi, whereas in North Carolina, dogs were monitored by owners. Method of pain assessment did not use a validated scale. Other than incisional swelling, complications were only monitored for the first 24 hours.

**Miller et al. (2018):** 

Population:	Shelter-owned male dogs between 2–5 months of age with a body weight 0.9–11.4 kg (median 3.6 kg) were recruited over a 12 month period for castration.
Sample size:	418 dogs.
Intervention details:	400 dogs underwent suture-less scrotal castration, and 18 dogs underwent traditional pre-scrotal castration. Dogs in both groups were anaesthetised with a combination of midazolam hydrochloride (0.55 mg/kg) and ketamine hydrochloride (5 mg/kg), after preoperative buprenorphine (0.015 mg/kg). Gas anaesthesia was maintained with isoflurane and 100% oxygen for the duration of the procedure. Scrotal and pre-scrotal areas were prepped with a 4% chlorhexidine solution and alcohol for both groups. A scrotal incision was made ventrally, and open or closed castration was performed at the discretion of the surgeon. The scrotal incision was closed with 1–2 drops of tissue glue. For the pre-scrotal procedure, a midline skin incision was made cranial to the scrotum and either an open or closed castration performed, as per the methods outlined for scrotal incision. Skin was closed with a single subcutaneous cruciate suture and the dermis closed with tissue glue. Duration of surgery was timed for the 18 dogs that underwent pre-scrotal castration procedures as well as for 18 dogs from the original 400 that underwent scrotal castration.
Study design:	Prospective single site clinical trial.
Outcome Studied:	Post-operative complications were monitored for 24 hours and included haemorrhage, pain, self-trauma, swelling, and dermatitis at the incision site. Incisional pain was assessed visually with and without gentle palpation. Duration of surgery was measured for two intervention groups.
Main Findings(relevant to PICO question):	Complication rates for open and closed castration methods were not significantly different (P=0.08). Post-operative complications reported: 9/400 (2.3%) had peri-incisional dermatitis, 4/400 (1%), had skin bruising, and 1/400 (0.3%) had swelling. Mean surgery time for the scrotal procedure was shorter (1.0 ± 0.2 minutes) than for the pre-scrotal procedure (3.5 ± 0.4 minutes) (P <0.001).
Limitations:	Complications were only monitored for 24 hours. No randomisation procedures described for the comparative study aspect (duration of surgery outcome). Lack of a positive control group to compare complication rates. Method of pain assessment did not use a validated scale.

## Appraisal, application and reflection

Castration in the male dog is a common surgical procedure with multiple indications including humane population control, modulation of certain undesirable behaviours (McGreevy et al., 2018), and as an intervention in the control of some health conditions caused by infectious, endocrine, testicular and epidydimal pathologies (Hamilton et al., 2014). It is conventionally taught using an incisional approach made cranial to the scrotum that avoids excessive tissue handling of the scrotal skin. Some authors have suggested that this approach, which is unique among domestic animal species, is due to a higher infection risk related to the canine scrotum resting on the ground during sitting (Wilson, 1975) or that male dogs are ‘scrotal conscious’ and prone to self-trauma (Howe, 2006). Since canine orchidectomy is widely taught and performed in companion animal practice and variations on the technique exist, evidence review is warranted to further refine a patient-centered approach for this routine procedure. In this critical appraisal of the literature, the evidence supporting the use of a scrotal incision to perform orchidectomy was evaluated against the traditional pre-scrotal approach.

Two North American studies met the inclusion criteria comparing patient outcomes after castration using the two different incisional access points. Post-operative recovery was assessed through observation of incisional swelling, haemorrhage, self-trauma, and pain. Since surgical duration is associated with incision infection risk (Eugster et al., 2004), the length of each procedure was also recorded and compared. Results from a randomised positive control multisite clinical trial conducted in 2015 found that the odds of self-trauma were reduced in dogs undergoing castration with a scrotal incision (OR: 0.51, P=0.04, 95% CI 0.27–0.97) in comparison to the positive control group, and that other complications observed were not attributable to differences in location of the skin incision (Woodruff et al., 2015). In a subsequent study by Miller et al. (2018), post-operative complications were recorded for 24 hours for 400 dogs after undergoing castration using a scrotal approach. Complications were reported as peri-incisional dermatitis in 9/400 (2.3%) dogs, as well as skin bruising in 4/400 (1.0%) dogs and incisional swelling in 1/400 (0.3%). In Britain, the reported rate of postoperative complications occurring at any time after canine castration is 10.83% (95% CI 8.51%–13.69%) (NASAN, 2021).

These two studies suggest that the risk of postoperative complications in the first 24 hours is at least no worse when a direct scrotal approach to orchidectomy is used and begin to unpick the assumption that the traditional pre-scrotal technique is safest. The protective effect of a scrotal incision against self-trauma reported by Woodruff et al. (2015) conflicts with historic assumptions related to sensitivity of the scrotal tissues. Moreover, both studies highlighted a shorter duration of surgery in dogs undergoing a scrotal castration approach. A methodology review suggested that increased surgical efficiency of direct scrotal access results from reduced time required to locate and exteriorise the gonads, as well as less time taken to close the incision, since the incision is normally left to heal by second intention or closed with tissue glue (DiGangi et al., 2016).

The preference for pre-scrotal access may be due to the cosmetic finish of a closed skin incision yielding minimal discharge, as this is generally considered to be more acceptable to pet owners since patients recover from surgery in the home. However, preventive wound management such as scrotal wrapping, adhesive dressing or a wound spray may lessen discharge (DiGangi et al., 2016), and the imperative to minimise complication risk for elective procedures in animals might one day support a shift in consensus on the most optimal access point for canine castration. Currently, however, several limitations prevent firm conclusions from being drawn from this critical appraisal of the evidence.

Only two studies met the inclusion criteria and both were conducted in the USA, where acceptance of companion animal neutering is generally very high. It is possible that patient outcomes observed in the included studies may not reflect those in other countries. Furthermore, use of multiple employees and / or private owners to observe patient recoveries as occurred in the assessed studies could have contributed to inconsistent observations. Neither study indicated that validated questionnaires or scoring systems were used in the assessment of subjective parameters, for example, the Glasgow Composite Measure Pain Scale to evaluate pain scores. Alongside inter-observer variability, differences in recovery setting (home recovery versus shelter) could also have influenced the likelihood of complication observation, since patients recovering in a home may receive more attentive care. Finally, further research is justified to evaluate postoperative complications that occur more than 24 hours after surgery, as well as trials conducted with larger sample sizes drawn from multiple sites.

This review provides support that frequently performed and routine elective procedures warrant periodic and critical review of underlying assumptions. Modern surgical techniques and perioperative care may improve outcomes of direct (scrotal) incisional approach in canine castration at least in the first 24 hours, but it remains unclear whether the advantages described in the papers reviewed would be maintained in the longer postoperative period.

## Methodology

**Search Strategy:** 

Databases searched and dates covered:	CAB Abstracts on OVID Platform 1973 to 2021 Week 47 PubMed accessed via the NCBI website 1920–December 2021
Search strategy:	CAB Abstracts: (dog* or canine* or canis).mp. or exp dogs/ (castrat* or neuter* or gonadectom* or steriliz* or sterilis*).mp. or exp sterilization/ or exp castration/ or exp gonadectomy/ ((surger* or surgical or incision* or approach or method).mp. or exp surgery/) adj3 ((pre-scrotal or pre-scrotal or prescrotum or scrotal or scrotum).mp. or exp scrotum/) 1 and 2 and 3 PubMed: #1 dog OR canine OR canis #2 castrate OR castration OR neuter OR gonadectomy OR sterilize OR sterilise #3 (surgery OR surgical OR incision OR approach OR method) AND (pre-scrotal OR pre-scrotal OR prescrotum OR scrotal OR scrotum) #4 #1 AND #2 AND #3
Dates searches performed:	10 Jun 2022

**Exclusion / Inclusion Criteria:** 

Exclusion:	Case studies or case series. Non-English language. Review article. Intervention not relevant to PICO.
Inclusion:	Clinical trial or observational study. English language. Canine species. Comparative study.

**Search Outcome:** 

Database	Number of results	Duplicates	Excluded – Not English or not accessible	Excluded – Review	Excluded – Other intervention	Total relevant papers
CAB Abstracts	23	0	5	2	16	0
PubMed	75	4	5	1	64	1
Hand Search	1	–	–	–	–	1
Total relevant papers when duplicates removed						2

## Acknowledgements

The author thanks the RCVS Library and Knowledge Services for assistance with the literature search. The author also gratefully acknowledges insightful discussions with Ukrainian colleagues, in particular Dr Kate Lutskevych, which eventually led to the development of this evidence appraisal.

## ORCID

Ariel Brunn: https://orcid.org/0000-0003-2084-6735

## Conflict of Interest

The author declares no conflicts of interest.
